# Informing the Development of an Interprofessional Geriatrics Core Curriculum: Stakeholder Engagement

**DOI:** 10.1093/geroni/igaf122.737

**Published:** 2025-12-31

**Authors:** Katharina Echt, Kathryn Nearing, Iriana Hammel, Elizabeth Chapman, Becky Powers, Sumi Misra, Beth Hogans

**Affiliations:** U.S. Department of Veterans Affairs, Brookhaven, Georgia, United States; University of Colorado Anschutz Medical Campus, Denver, Colorado, United States; Miami VA Medical Center, Miami, Florida, United States; William S. Middleton Memorial Veterans’ Hospital, Madison, Wisconsin, United States; The University of Texas Health Science Center at San Antonio, San Antonio, Texas, United States; Vanderbilt University Medical Center, Nashville, Tennessee, United States; Johns Hopkins School of Medicine, Baltimore, Maryland, United States

## Abstract

Early-stage, formative solicitation of stakeholder needs and preferences can inform curriculum development that is responsive and fosters a sense of co-ownership. Clinical discipline preceptors are key collaborators in the ultimate implementation of an interprofessional core curriculum in geriatrics with their respective health professions trainees. This presentation describes the formative environmental scan and subsequent stakeholder engagement process to identify 1) core geriatric knowledge needed by interprofessional trainees, 2) gaps in geriatric education across discipline-specific programs, and 3) perceived barriers and delivery/modality preferences. The Geriatric Interprofessional Core Curriculum development team conducted an environmental scan to identify existing interprofessional core curricula that provide essential knowledge in best practices for caring for older adults. The team identified multiple relevant and validated resources and iteratively discussed strengths and limitations of each. Few were sufficiently inclusive to meaningfully engage the range of disciplines and levels of professional preparation represented in the GRECC health professions training program. The Geriatric 5M’s Framework (What Matters, Mentation, Medication, Mobility, Multicomplexity) provided an interprofessional scaffold that, with preceptors’ subject matter expertise, facilitated curriculum development. Accordingly, five roundtable discussions were held, each focusing on one of the 5Ms. A total of 49 interprofessional educators were engaged and asked to identify core geriatric training needs, discuss preferred curriculum modality and potential barriers to implementation. Thematic analysis of transcripts and detailed session notes yielded a set of priority content topics, as well as implementation preferences regarding usable formats and delivery, in support of the next phase of the development process.

